# The efficacy of Link N as a mediator of repair in a rabbit model of intervertebral disc degeneration

**DOI:** 10.1186/ar3423

**Published:** 2011-07-25

**Authors:** Fackson Mwale, Koichi Masuda, Rajeswari Pichika, Laura M Epure, Tomoaki Yoshikawa, Aseem Hemmad, Peter J Roughley, John Antoniou

**Affiliations:** 1 Division of Orthopaedic Surgery, McGill University, 1650 Cedar Avenue, Montreal, QC, Canada, H3G 1A4; 2 Lady Davis Institute for Medical Research, SMBD-Jewish General Hospital, 3755 Chemin de la Cote Ste-Catherine, Montreal, QC, Canada, H3T 1E2; 3 Department of Orthopaedic Surgery, School of Medicine, University of California, San Diego, 9500 Gilman Drive, Mail Code 0863, La Jolla, CA 92093-0863, USA; 4 Genetics Unit, Shriners Hospitals for Children, 1529 Cedar Avenue, Montreal, QC, Canada, H3G 1A6

## Abstract

**Introduction:**

Intervertebral disc (IVD) degeneration is associated with proteolytic degradation of the extracellular matrix, and its repair requires both the production of extracellular matrix and the downregulation of proteinase activity. These properties are associated with several growth factors. However, the use of growth factors in clinical practice is limited by their high cost. This cost can be circumvented using synthetic peptides, such as Link N, which can stimulate the synthesis of proteoglycan and collagen by IVD cells *in vitro*. The purpose of the present study was to evaluate the effect of Link N *in vivo *in a rabbit model of IVD degeneration.

**Methods:**

New Zealand white rabbits received annular puncture in two lumbar discs. Two weeks after puncture, both punctured discs of each rabbit were injected with either Link N or saline. After 2 weeks, nine rabbits were euthanized and the annulus fibrosus (AF) and nucleus pulposus (NP) of Link N-injected and saline-injected IVDs were removed and used to prepare total RNA. Following reverse transcription, quantitative PCR was performed for aggrecan, COL2A1, COL1A1, ADAMTS-4, ADAMTS-5 and MMP-3. After 12 weeks, 19 rabbits were euthanized and the injected IVDs were removed for biochemical and histological analysis. Proteinase K digests were analyzed for DNA and sulfated glycosaminoglycan content. Disc height was monitored radiographically biweekly.

**Results:**

Following needle puncture, disc height decreased by about 25% over 2 weeks, and was partially restored by Link N injection. Puncture of the IVD resulted in a trend towards decreased proteoglycan content in both the NP and AF, and a trend towards partial restoration following Link N injection, although under the time course used this did not achieve statistical significance. Link N did not alter the DNA content of the discs. Link N injection led to a significant increase in aggrecan gene expression and a significant decrease in proteinase gene expression in both the NP and AF, when compared with saline alone.

**Conclusions:**

When administered to the degenerate disc *in vivo*, Link N stimulated aggrecan gene expression and downregulated metalloproteinase expression, and there was a trend towards increased proteoglycan content of the disc, in both the NP and AF. These are features needed for any agent designed to stimulate disc repair. In principle, therefore, Link N supplementation could be an option for treating disc degeneration.

## Introduction

Low back pain is an insidious disorder that, by age 70, affects about 60% of the population. Although the etiology of low back pain is often unclear, it is believed that intervertebral disc (IVD) degeneration plays a major role [1,2]. While present management of disc pathology has been focused on symptoms associated with degeneration, fewer studies have been devoted to disc regeneration. Current surgical procedures such as disc excision and vertebral fusion [3] lead to relief of pain in the short term, but they alter the biomechanics of the spine, leading to further degeneration of surrounding tissue and discs at adjacent levels. Newer treatment methods such as artificial disc implants are controversial, as their insertion partially disrupts the disc structure and may eventually destabilize the motion segment. Procedures to invoke biological repair of the degenerate disc could help resolve these concerns.

Discs allow bending and twisting of the spine whilst resisting compression from gravity and muscle action [4]. The discs are thought to resist compressive forces by their high content of the proteoglycan aggrecan, which interacts with hyaluronate to produce large proteoglycan aggregates, with each interaction being stabilized by the further interaction of a link protein [5,6]. The proteoglycan aggregates induce a high swelling pressure in the nucleus pulposus (NP) that is balanced by tensile forces produced in the collagen network of the annulus fibrosus (AF). Disc degeneration is associated with biochemical alterations in the composition and structure of the extracellular matrix (ECM) due to depleted synthesis and increased degradation, with aggrecan being particularly susceptible to proteolytic damage and loss. While poor IVD nutrition may be a major contributor to disc degeneration, biomechanical [7-9], biochemical [10-15] and genetic [16,17] influences may also play a role in some individuals. The degenerate discs have little capacity for endogenous repair because of their lack of blood vessels and poor nutrition. Inducing repair of disc tissue may be possible, however, as the use of chymopapain to degrade the degenerate NP can stimulate new ECM formation [18,19], although not consistently. Cell or growth factor therapies have also recently been suggested to induce IVD repair [20-24].

The stimulation of repair in the degenerate IVD requires both the production of ECM and the downregulation of proteinase activity. Matrix synthesis is associated with several growth factors, including transforming growth factor beta and bone morphogenetic protein 7 [25-28] However, one problem with the use of growth factors in clinical practice is their high cost. In principle this can be circumvented using synthetic peptides, which are relatively cheap to produce. One peptide with the ability to stimulate ECM synthesis and therefore possessing growth factor-like properties is Link N [29-31]. Link N (DHLSDNYTLDHDRAIH) is the N-terminal peptide of the link protein that stabilizes the proteoglycan aggregates. This peptide is generated *in vivo *by proteolytic degradation during tissue turnover [32]. Previous studies showed that Link N can stimulate synthesis of proteoglycans and collagens in articular cartilage [29,33,34]. Link N can also preferentially stimulate the synthesis of proteoglycan over collagen by bovine IVD cells *in vitro*, without any effect on cell division [30]. To date, however, there have been no reports on the effect of Link N on the IVD *in vivo*. The purpose of the present study was to determine the effect of intradiscally administered Link N on disc cell survival and whether Link N can stimulate ECM production and downregulate proteinase production in a rabbit annular needle puncture model of IVD degeneration.

## Materials and methods

### Synthesis of Link N

Link N was synthesized with a purity > 98% by CanPeptide Inc. (Pointe-Claire, QC, Canada).

### Rabbit annular puncture model and Link N injection

Thirty-eight New Zealand white rabbits weighing approximately 3.5 kg (5 to 6 months old) were used in the present study with the approval of the Institutional Animal Care and Use Committee. Twenty-eight rabbits received the annular puncture with an 18-gauge needle and a 5 mm depth of puncture on two noncontiguous discs (L2/L3 and L4/L5), as previously described [22,35]. Two weeks after the initial puncture, both previously punctured discs of each rabbit were injected either with saline (10 μl per disc) or with Link N (100 μg in 10 μl saline per disc) through their anterolateral surfaces into the center of the NP using a 26-gauge needle. Ten rabbits, which were not punctured and remained untreated, were used as a control group for biochemical analysis and histologic evaluation.

### Radiographic analysis of disc height

Radiographs were taken using a digital radiography system (resolution 71 μm; NAOMI, Nagano, Japan) after administration of ketamine hydrochloride (25 mg/kg) and acepromazine maleate (1 mg/kg), at biweekly intervals up to 12 weeks after the puncture. The preoperative radiograph, which was imaged 2 or 3 days before the surgery, was always used as a baseline measurement. A strict protocol was used to obtain optimal images for the image analysis, as previously described [35]. To decrease the error from axial rotation of the spine and from beam divergence, radiographs were repeated until a reasonable alignment of transverse processes (within one-half width of processes as a deviation) was achieved on each animal in the lateral decubitus position with the beam centered 4 cm from the rabbit iliac crest.

Analysis of disc height was performed, as previously described, with further normalization to the L3/L4 disc [35]. All radiograph images were digitized and independently analyzed using a custom program for MATLAB software (Natick, MA, USA) by an orthopedic researcher who was blinded to the treatment groups. The average of the disc height index (DHI) was calculated as a ratio of the average anterior, middle, and posterior IVD space measurements to the average of the adjacent vertebral body heights. Changes in the DHI of injected discs were expressed as a percentage, and were calculated as previously described [35]:

The %DHI was further normalized using the nonpunctured L3/L4 level as a control in order to account for the vertebral body growth:

Using this technique, the intraobserver error (percentage coefficient of variance = 3.13%) and interobserver error (percentage coefficient of variance = 9.6%) of DHI measurements have been reported to be minimal [35].

### RNA extraction and gene expression analyses

Nine rabbits were euthanized 2 weeks after either Link N or saline injection, and the injected IVDs (L2/L3 and L4/L5) from both experimental groups were removed from each lumbar spine for gene expression analysis (Table 1). After disc excision, the NP was bluntly separated from the AF. Each tissue was snap-frozen and pulverized using a Cryopress (Microtech Ltd, Tokyo, Japan). Total RNA was extracted from the pulverized tissues using an RNeasy mini kit (Qiagen, Valencia, CA, USA). After extraction, RNA was quantified using a Nanodrop N-1000 spectrophotometer (Fisher Scientific, Wilmington, DE, USA). One microgram of total RNA from each AF or NP was reverse-transcribed into cDNA using the Superscript™ First Strand cDNA synthesis kit (Invitrogen, Carlsbad, CA, USA). Two microliters of cDNA were amplified using gene-specific primers (Table 2). The PCR was carried out by denaturing at 95°C for 15 minutes, followed by annealing at 58 to 65°C for 30 seconds (Table 2), then extension at 72°C, repeated for 55 cycles using a SYBR green kit (Qiagen). All PCR reactions were carried out using a Roche Light Cycler (Roche Diagnostics, Indianapolis, IN, USA), and mRNA expression was quantified using a standard curve generated with cloned cDNA plasmids containing target PCR products. The expression of the target genes was first normalized to GAPDH expression levels, and then the expression of the Link N-treated discs was normalized to saline-treated discs.

**Table 1:** Number of animals and discs used in the study

	2 weeks after injection		12 weeks after injection		Total	
	Rabbits (*n*)	Discs (*n*)	Rabbits (*n*)	Discs (*n*)	Rabbits (*n*)	Discs (*n*)
Gene expression						
Saline	4	8			9	18
Link N	5	10				
Biochemistry						
Control			6	12		
Saline^a^			6	12	19	38
Link N^a^			7	14		
Histology						
Control			4	8		
Saline			3	6	10	20
Link N			3	6		

^a^These rabbits were also used for biweekly X-ray measurements of disc height.

**Table 2:** Oligonucleotide primers used to assess gene expression

Gene	Primer sequence (5' to 3')	Annealing temperature (°C)
Aggrecan	Forward: GAGGTCGTGGTGAAAGGTGT	60
	Reverse: GTGTGGATGGGGTACCTGAC	
COL1A1	Forward: AGGGCCAAGACGAAGACATC	62
	Reverse: AGATCACGTCATCGCACAACA	
COL2A1	Forward: CAACACTGCCAACGTCCAGAT	62
	Reverse: CTGCTTCGTCCAGATAGGCAAT	
MMP-3	Forward: TTTTGGCCATCTCTTCCTTCA	65
	Reverse: TGTGGATGCCTCTGGGTATC	
ADAMTS-4	Forward: GACCTTCCGTGAAGAGCAGTGT	58
	Reverse: CCTGGCAGGTGAGTTTGCAT	
ADAMTS-5	Forward: CCTGGCAGGTGAGTTTGCAT	60
	Reverse: GGAGAACATATGGTCCCAACGT	
GAPDH	Forward: ACTCTGGCAAAGTGGATG	60
	Reverse: TCCTGGAAGATGGTGATG	

### Biochemical analysis

Thirteen rabbits were euthanized 12 weeks after either Link N or saline injection, and the injected IVDs (L2/L3 and L4/L5) from both experimental groups were removed from each lumbar spine for biochemical analysis (Table 1). Twelve discs from six additional control rabbits were also analyzed. After disc excision, the NP was bluntly separated from the AF. All specimens were weighed (wet weight) and digested with proteinase K at 56°C for 48 hours. The content of DNA in the digest was analyzed by a fluorometric DNA assay using PicoGreen [36]. The data were normalized to a per disc basis. Wet weight basis normalization was avoided because of disc swelling. The proteinase K digests were also analyzed for proteoglycan (predominantly aggrecan) as sulfated glycosaminoglycan using the 1,9-dimethylmethylene blue dye-binding assay [37], and for hyaluronate using a competitive hyaluronate-binding assay [38].

### Histology

Six rabbits were euthanized 12 weeks after either Link N or saline injection, and the injected IVDs (L2/L3 and L4/L5) from both experimental groups were removed from each lumbar spine for histology (Table 1). Eight discs from four additional control rabbits were also evaluated. The IVDs were excised and fixed in 10% neutral buffered formalin solution, decalcified in Plank Rychlo solution (Cal-Ex* II Fixative/Decalcifier, Fisher Scientific, Wilmington, DE, USA), dehydrated in a graded series of ethanol (70%, 90% and 99%), and processed individually for paraffin embedding, as previously described [22]. The paraffin blocks were sectioned longitudinally using a microtome to give 5 μm sections. The paraffin sections were dewaxed and stained with Safranin O to detect proteoglycan (predominantly aggrecan).

### Statistical analysis

Statistical analysis was performed using the Statview (version 5.0; SPSS, Chicago, IL, USA) program package. Differences between the Link N-injected and saline-injected groups were assessed with one-way repeated analysis of variance and Fisher protected least significant difference as a *post hoc *test.

## Results

### Radiographic assessment

**Figure 1:**
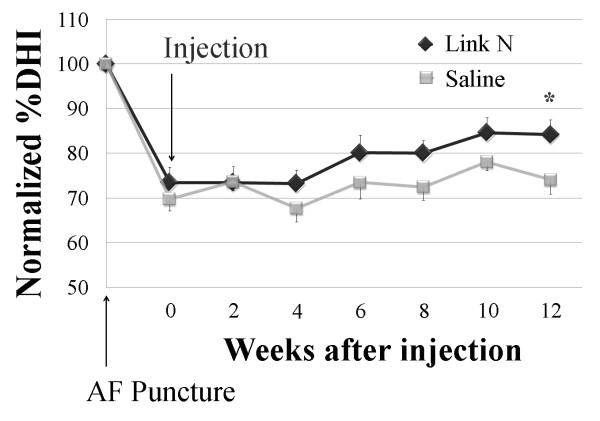
**Changes in intervertebral disc height index after annulus fibrosus puncture and injection**. Changes in the intervertebral disc height index (DHI) after the annulus fibrosus (AF) puncture and saline or Link N injections. The DHI was measured at 2-week intervals to quantify changes in disc height. Values represent the mean normalized %DHI ± standard deviation of Link N-injected and saline-injected discs at each time point. By 4 weeks after the Link N injection, the mean normalized %DHI of injected discs in the Link N group was higher than in the saline group. This difference was maintained during the next 8 weeks. By 12 weeks after the Link N injection, the mean normalized %DHI of injected discs in the Link N group was significantly higher than in the saline group (*P < 0.05).

The initial AF puncture with an 18-gauge needle to initiate disc degeneration was performed identically in both the saline and Link N groups. Following needle puncture the normalized %DHI decreased by about 25% over the next 2 weeks compared with the baseline DHI values obtained before puncture (Figure 1). By 4 weeks after the Link N injection, the mean normalized %DHI of the injected discs in the Link N group was higher than in the saline group. This difference in normalized %DHI was maintained for the following 8 weeks with a statistically significant increase after 12 weeks (*P *< 0.05).

### Aggrecan expression

**Figure 2:**
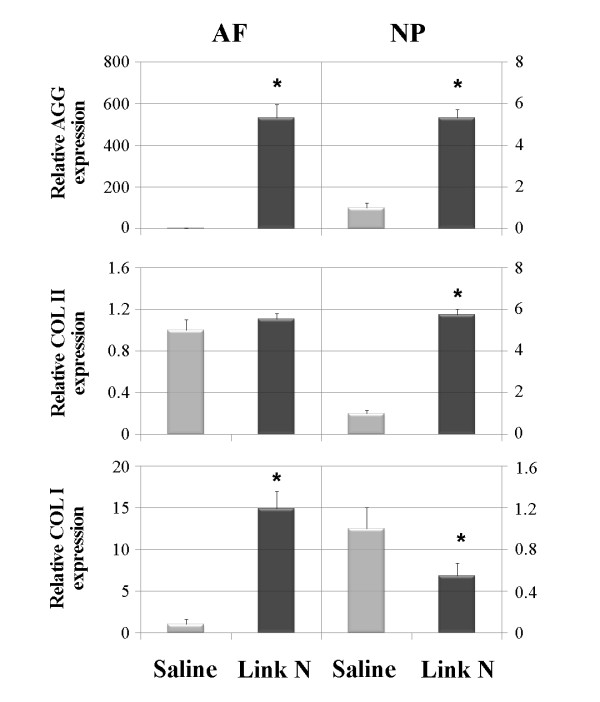
**Changes in aggrecan, type II collagen and type I collagen gene expression**. Changes in aggrecan (AGG), type II collagen COL2A1 (COL II) and type I collagen COL1A1 (COL I) gene expression of the annulus fibrosus (AF) and nucleus pulposus (NP) 2 weeks after Link N or saline injections. Gene expression was measured by RT-PCR. GAPDH was used as a housekeeping gene and served to normalize the results. Values represent the mean ± standard deviation of Link N-injected discs normalized to saline-treated discs (*P < 0.001).

Aggrecan message levels were examined following Link N treatment because it is the major contributor to the sulfated glycosaminoglycan content of the tissue and hence is responsible for tissue swelling and function. Furthermore, aggrecan loss is a feature of disc degeneration, and its replacement is essential for repair. Link N injection led to a significant increase (*P *< 0.001) in aggrecan gene expression in both the AF and NP, when compared with saline alone (Figure 2).

### Collagen expression

Collagen message levels were examined following Link N treatment because the collagen fibrils allow the IVD to entrap the proteoglycan aggregates as well as provide tensile strength to the tissue. Link N injection led to a significant increase (*P *< 0.001) in type II collagen (COL2A1) gene expression in the NP, when compared with saline alone (Figure 2). Although there was a slight increase in COL2A1 message in the AF, this was not significant (*P *= 0.36). In contrast, Link N injection led to a significant increase (*P *< 0.001) in type I collagen (COL1A1) gene expression in the AF but a significant decrease in the NP (*P *< 0.01) (Figure 2).

### Proteinase expression

**Figure 3:**
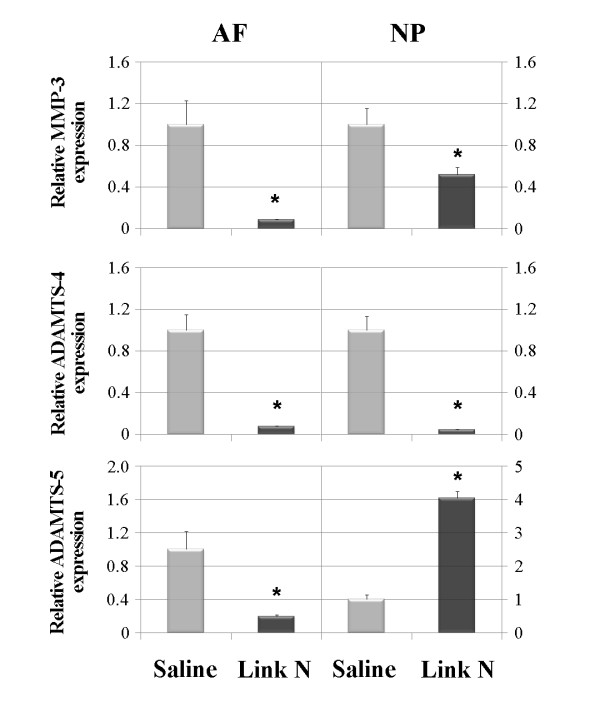
**Changes in MMP-3, ADAMTS-4, and ADAMTS-5 gene expression**. Changes in MMP-3, ADAMTS-4, and ADAMTS-5 gene expression of the annulus fibrosus (AF) and nucleus pulposus (NP) 2 weeks after Link N or saline injections. Gene expression was measured by RT-PCR. GAPDH was used as a housekeeping gene and served to normalize the results. Values represent the mean ± standard deviation of Link N-injected discs normalized to saline-treated discs (*P < 0.001).

Since metalloproteinases, particularly members of the ADAMTS and matrix metalloproteinase (MMP) families, are major contributors to aggrecan degradation and loss in disc degeneration, MMP-3, ADAMTS-4 and ADAMTS-5 message levels were also examined, as their suppression is essential for disc repair. Link N injection led to a significant decrease in MMP-3 (*P *< 0.001) and ADAMTS-4 (*P *< 0.001) gene expression in both the AF and NP tissues, when compared with saline alone (Figure 3). In contrast, Link N injection led to a significant decrease in the gene expression of ADAMTS-5 in the AF (*P *< 0.001) but an increase in NP tissues (*P *< 0.001) when compared with saline alone (Figure 3).

### DNA content

**Figure 4:**
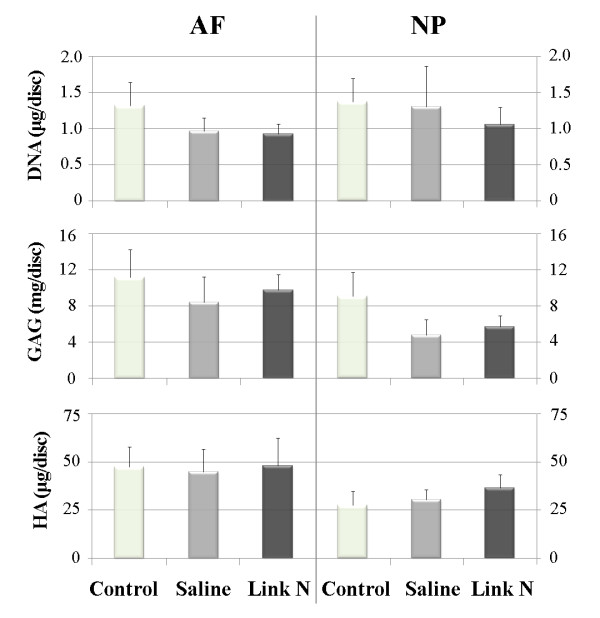
**Changes in DNA, proteoglycan (sulfated glycosaminoglycan) and hyaluronic acid content**. Changes in DNA, proteoglycan (sulfated glycosaminoglycan (GAG)) and hyaluronic acid (HA) contents of the annulus fibrosus (AF) and nucleus pulposus (NP) in nonpunctured control discs and 12 weeks after Link N or saline injections. Values represent the mean ± standard deviation of Link N and saline-injected discs and nonpuncture discs at 12 weeks post injection. There is a trend towards Link N stimulating GAG and HA production but it is statistically not significant (repeated analysis of variance, *P *> 0.8). The injection of Link N did not affect the DNA contents in the NP and AF.

DNA was analyzed in order to determine the effect of Link N on cell proliferation. AF puncture decreased the DNA content of the discs and neither subsequent saline nor Link N injection caused an increase in the DNA content 12 weeks after injection (Figure 4). Similar DNA contents were observed for the NP although the saline group appeared to be slightly higher than the Link N-injected group. These changes were not statistically significant.

### Proteoglycan content

Puncturing the IVD led to a decrease in proteoglycan content in both the NP and the AF after 12 weeks in saline-treated discs (Figure 4). Treatment with Link N for 12 weeks following initiation of degeneration resulted in increased proteoglycan content in both the NP and AF by about 20%, when compared with saline-treated discs. Similar changes were observed when the sulfated glycosaminoglycan/DNA ratio was measured (data not shown). However, these trends were not statistically significant.

### Hyaluronic acid content

The hyaluronic acid content was also monitored because proteoglycan loss can also be associated with hyaluronic acid degradation and loss. Hyaluronic acid levels were higher in the AF than the NP, but levels were not affected by treatment (Figure 4). These changes were not statistically significant.

### Histology

**Figure 5:**
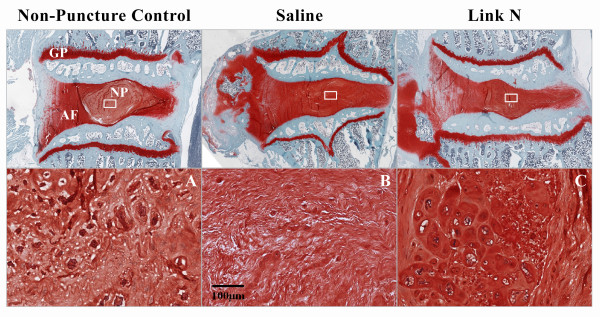
**Histologic changes after injection of saline or Link N**. Safranin O-stained sections of control (nonpunctured), saline-treated, and Link N-treated discs comparing the degree of degeneration. In comparison with the saline-treated group, the Link N group displayed a restoration of the nucleus pulposus (NP) with the presence of intensely stained matrix and large cells typical of a control nucleus pulposus. AF, annulus fibrosus; GP, growth plate.

In order to examine the effect of Link N on the IVD matrix, discs were studied histologically by Safranin O staining, which detects the chondroitin sulfate chains of aggrecan. Red Safranin O staining was present in the AF and the vertebral growth plates in the control group (Figure 5a). The NP was rounded, and distinct from the AF. The Safranin O-stained histologic sections in the saline-treated group showed signs of early degeneration, with the NP being indistinct from the AF and with wavy fibrocartilage lamellae and associated fibrochondrocytes being apparent (Figure 5b). In contrast, in the Link N-treated group, the NP consisted of numerous large, vacuolated cells and smaller chondrocyte-like cells that often appeared in clusters (Figure 5c).

## Discussion

Previous studies have shown that Link N can act as a growth factor and stimulate the synthesis of proteoglycans and collagens in articular cartilage [29,32-34], as well as bovine IVD cells *in vitro *[30]. The present data indicate that there is a trend towards Link N stimulating the proteoglycan content when it is administered to the degenerate rabbit disc *in vivo*. This stimulation occurs in both the NP and AF of the disc and in the absence of any effect on cell division. In addition to stimulating aggrecan gene expression, Link N is also able to downregulate metalloproteinase gene expression in the degenerate disc. These are features needed for any agent designed to stimulate disc repair. In principle, therefore, Link N supplementation could be a viable option for treating disc degeneration during its early stages while the AF is intact. An intact AF is essential for optimal repair in order to prevent the protrusion of the NP due to the increased swelling potential associated with proteoglycan accumulation.

One exception to the downregulation of proteolytic activity by Link N was observed in the NP for ADAMTS-5, where gene expression was increased. At first sight this is not what one would predict for repair, and it remains to be seen whether increased expression is detrimental or not. Repair involves remodeling of the disc ECM, however, and remodeling involves proteolysis. Hence there is no need for a complete absence of proteolysis during repair, as long as the matrix synthesis exceeds turnover.

The proteoglycan changes observed with Link N are similar to those reported after 12 weeks when the same concentration of osteogenic protein-1 (OP-1) was injected into the degenerate rabbit disc [22]. The injected discs in the Link N group also display a similar trend in disc height changes to that produced by OP-1 [22]. Link N thus appears to be effective at stimulating repair of the IVD *in vivo*. One major advantage of Link N over a growth factor such as OP-1 for therapeutic use is the large saving in cost. Link N costs $750 for 50 mg, which is about $1.5 per 100 μg injected in the rabbit. In contrast, OP-1 costs $600 per 100 μg. Link N therefore represents a potential economical therapeutic agent with beneficial effects. However, a longer time frame following Link N administration will be necessary to truly prove the value of Link N and to determine whether its beneficial effect can be sustained. In addition, it is not clear to what degree the proteoglycan content of the disc must be improved in order to influence disc function, and it may be necessary to use additional techniques to ensure functional repair.

Despite the significant impairment associated with degenerative disc disease, a clear understanding of its pathogenesis is still lacking. Currently, no animal model parallels the complex nature of human disc degeneration due to variation in disc composition and time course of progression [39-41]. An animal model, however, is essential to test the efficiency of disc repair. Several models of induced or spontaneous disc degeneration have been used in the past, but each type of model has limitations [39-41]. One issue is the difficulty of using small animals such as rodents because of the small size and volume of their discs. In addition, the NPs cells remain notochordal in the adult, unlike the human and many large animal models. On the other hand, larger animals, such as dog and sheep, are very expensive and not appropriate to establish new experimental conditions, although they are useful for testing approaches established in smaller animal models. The rabbit IVD aspiration model used in the present study represents a compromise between the size of the animal, disc composition, and the cost of the experiments. It has been used for its reproducibility in creating mild degeneration and utility for evaluating treatment efficacy [21,35].

For biological repair of the degenerative IVD to be successful in humans, it will probably be essential to perform treatment early in the degenerative process, before major damage to the collagen framework has occurred. It will therefore be essential to determine whether Link N can stimulate repair of the degenerated human IVD in Thomson grade 2 and grade 3 discs [42]. While such treatment may not be able to fully repair disc degeneration, it should be able to retard its progression and so delay the need for more aggressive surgical intervention. To better understand the effect of Link N, further studies on the mechanisms of action of this peptide, including the receptors with which it interacts and the intracellular signaling pathways that it transduces, will be necessary. Ultimately, these studies should lead to clinical trials on the repair of the NP in the degenerated IVD using Link N.

## Conclusions

Link N can stimulate proteoglycan production *in vivo *in both the NP and AF when it is administered to the degenerate disc. Interestingly, the changes in proteoglycan synthesis with Link N are similar to those reported previously with the same concentration of OP-1. In addition to stimulating the synthesis of aggrecan, Link N is also able to downregulate metalloproteinase expression in the degenerate disc. These are features needed for any agent designed to stimulate disc repair. In principle, therefore, Link N supplementation could be a viable option for treating disc degeneration. However, a longer time frame following Link N administration will be necessary to truly prove the therapeutic value of Link N and show that it can actually restore the functional properties of the disc. If Link N administration is to be successful in humans, it will have to be performed early in the degenerative process before major damage to the collagen framework has occurred. While such treatment may not be able to fully repair disc degeneration, it should be able to retard its progression and so delay the need for more aggressive surgical intervention.

## Abbreviations

ADAMTS: a disintegrin and metalloprotease with thrombospondin-like repeats; AF: annulus fibrosus; COL1A1: type I collagen alpha 1; COL2A1: type II collagen alpha 1; DHI: disc height index; ECM: extracellular matrix; GAPDH: glyceraldehyde 3-phosphate dehydrogenase; IVD: intervertebral disc; MMP: matrix metalloproteinase; NP: nucleus pulposus; OP-1: osteogenic protein-1; PCR: polymerase chain reaction; RT: reverse transcription.

## Competing interests

The authors declare that they have no competing interests.

## Authors' contributions

FM conceived this study and wrote the manuscript. KM participated in the design of the study and performed the surgery. LME performed the biochemical analysis and statistical analysis, and was involved in preparation of the manuscript. RP, TY and AH performed data acquisition and statistical analysis. PJR participated in the design of the study and interpretation of the data, and revised the manuscript. JA made substantial contributions to the study design and revised the manuscript. All authors read and approved the final manuscript.
